# Association between non-high-density lipoprotein cholesterol and haemorrhagic transformation in patients with acute ischaemic stroke

**DOI:** 10.1186/s12883-020-1615-9

**Published:** 2020-02-07

**Authors:** Yanan Wang, Quhong Song, Yajun Cheng, Chenchen Wei, Chen Ye, Junfeng Liu, Bo Wu, Ming Liu

**Affiliations:** Department of Neurology, West China Hospital, Sichuan University, No. 37, Guo Xue Xiang, Chengdu, 610041 China

**Keywords:** Non-high-density lipoprotein cholesterol, Haemorrhagic transformation, Acute ischaemic stroke

## Abstract

**Background:**

It is unclear whether non-high-density lipoprotein cholesterol (Non-HDL-C) is associated with haemorrhagic transformation (HT) after acute ischaemic stroke (AIS). We aimed to explore the association between Non-HDL-C and HT, as well as compare the predictive values of Non-HDL-C and low-density lipoprotein cholesterol (LDL-C) for HT.

**Methods:**

We consecutively enrolled AIS patients within 7 days of stroke onset. Participants were divided into four categories according to quartiles of Non-HDL-C. HT was assessed by follow-up brain imaging. We assessed the association between Non-HDL-C, LDL-C and HT in multivariate logistic regression analysis.

**Results:**

A total of 2043 patients were included, among whom 232 were identified as HT. Compared with the highest quartiles, the first, second and third quartiles of Non-HDL-C were associated with increased risk of HT (adjusted odds ratios [ORs] 1.74 [95% confidence interval [CI] 1.09–2.78], 2.01[95% CI 1.26–3.20], and 1.76 [95% CI 1.10–2.83], respectively, *P* for trend = 0.024). Similar results were found for LDL-C. There was significant interaction between Non-HDL-C and age (*P* for interaction = 0.021). The addition of Non-HDL-C and LDL-C to conventional factors significantly improved predictive values [Non-HDL-C, net reclassification index (NRI) 0.24, 95%CI 0.17–0.31, *P* < 0.001; LDL-C, NRI 0.15, 95%CI 0.08–0.22, *P* = 0.03].

**Conclusions:**

Low Non-HDL-C was associated with increased risks of HT. In addition, Non-HDL-C has similar effects as LDL-C for predicting HT.

## Background

Haemorrhagic transformation (HT) is a common complication after acute ischaemic stroke (AIS), occurring in about 10–40% of patients [1]. The presence of HT may contribute to poor outcome in stroke patients [2]. A number of factors associated with HT have been reported, including age, stroke severity, atrial fibrillation and thrombolysis.

Although dyslipidaemia is known as an important risk factor of stroke [3], the association between the serum lipid levels and HT has not been well established. Prior studies have stated that low level of low-density lipoprotein cholesterol (LDL-C) could increase HT in patients with AIS [4–6], whereas the relationship between non-high-density lipoprotein cholesterol (Non-HDL-C) and HT is still not clear. As a composite marker, Non-HDL-C includes the triglyceride-rich lipoproteins such as chylomicrons, LDL, very-low-density lipoproteins, and their remnants [7]. Recent studies demonstrated that Non-HDL-C was more strongly associated with cardiovascular diseases than LDL-C [8–10]. However, whether Non-HDL-C is superior to LDL-C for predicting HT has not been studied. Therefore, we aimed to explore the association between Non-HDL-C and HT, as well as compare the predictive values of Non-HDL-C and LDL-C for HT in patients with AIS.

## Methods

### Study population

We consecutively enrolled ischaemic stroke patients within 7 days of stroke onset between January 2016 and September 2018 based on the Chengdu Stroke Registry Database, which has been described in details [11]. All patients were clinically diagnosed as ischaemic stroke based on the World Health Organization criteria, [12] and finally confirmed by computed tomography (CT) or magnetic resonance imaging (MRI) scan. Patients were not eligible if they: (1) were diagnosed with HT based on the initial head CT on admission, or (2) did not undergo later CT or MRI scan; or (3) lacked lipid profile test within 24 h after admission.

### Data collection

Baseline information including patients’ demographics, stroke severity on admission, medical history, current smoking, alcohol consumption, systolic blood pressure (SBP), diastolic blood pressure (DBP), blood glucose, lipid parameters, the Trial of ORG 10172 in Acute Stroke Treatment (TOAST) classification, thrombolysis and thrombectomy were collected. Medical history contained hypertension, diabetes mellitus, hyperlipidaemia and atrial fibrillation. Stroke severity on admission was evaluated using the National Institutes of Health Stroke Scale (NIHSS) [13].

### Lipid parameters

Blood samples were collected within 24 h after hospital admission, and serum lipid parameters, including total cholesterol (TC), triglycerides (TG), HDL-C and LDL-C, were tested in the Department of Laboratory Medicine, West China Hospital. Non-HDL-C levels were determined by subtracting serum HDL-C levels from total cholesterol [14].

### Assessment of HT

HT was defined as haemorrhage within the infarcted area or parenchyma hemorrhage outside the infarct zone that was present on a second CT or MRI (usually within 7 ± 2 days after admission), but not on head CT or MRI on admission, based on the European Cooperative Acute Stroke Study II criteria [15]. Additionally, HT was classified into symptomatic or no symptomatic HT group based on whether patients experienced any neurological deterioration [16]. HT was identified separately by two researchers (YW and QS), and a third researcher (CW) was consulted when a disagreement occurred.

### Statistical analysis

Participants were divided into four categories according to quartiles of Non-HDL-C. Continuous variables are described as means with the standard deviations or median with interquartile ranges, and categorical variables are presented as frequencies with percentages. Differences in continuous data were assessed using Student’s t test, ANOVA test, the Mann–Whitney U test or the Kruskal–Wallis test and differences in categorical data were assessed using the chi-squared test or Fisher’s exact test. Univariate analysis was carried out to identify possible risk factors for HT. We further performed multivariate logistic regression analysis to assess the association between Non-HDL-C or LDL-C and HT. The odds ratio (OR) and 95% confidence interval (CI) was calculated. We created two models. Model 1 adjusted for age and sex. Model 2 adjusted for other potential confounding variables besides age and sex on model 1. Multivariable spline regression model was used to test nonlinear relationship between LDL-C, Non-HDL-C and HT. In addition, C statistics and net reclassification index [17] were calculated to evaluate the predictive value of adding Non-HDL-C or LDL-C to conventional risk factors model. In addition, we performed stratified analyses to explore potential indicators that may modify the relationship between Non-HDL-C and HT. The significance of interaction was tested by the likelihood ratio test. All statistical analyses were performed using SPSS 22.0 (IBM, Chicago, IL, USA), R (http://www.R-project.org, The R Foundation) and EmpowerStats (http://www.empowerstats.com, X&Y Solutions, Inc., Boston, MA, USA). Two-sided values of *P* < 0.05 were considered statistically significant.

## Results

### Baseline characteristics

**Fig. 1:**
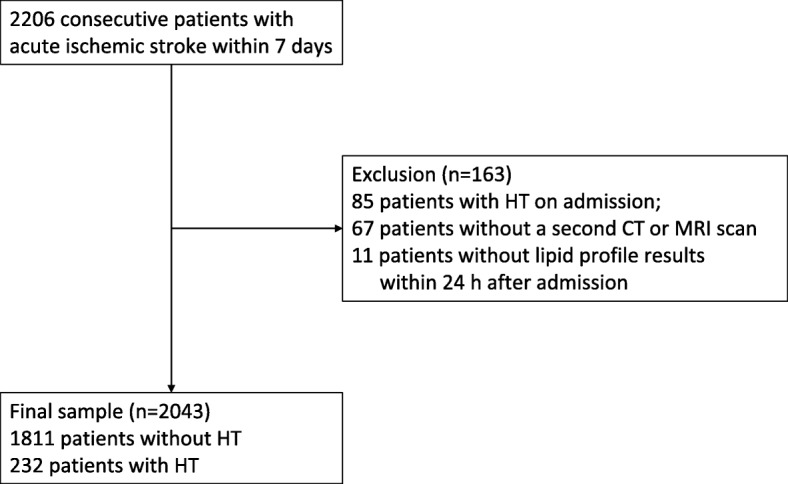
Study patients flow chart. HT: haemorrhagic transformation; CT: computed tomography; MRI: magnetic resonance imaging

In all, 2206 consecutive patients with ischaemic stroke within 7 days were admitted to our hospital during the study period and 2043 patients were included in this study (Fig. 1). Of these 2043 patients, the mean age was 65 ± 14 years, and 63.1% were males.

Demographic and clinical characteristics of participants based on Non-HDL-C quartiles are summarised in Table 1. Non-HDL-C levels ranged from 0.59 to 10.03 mmol/L (mean value, 3.14 mmol/L). Patients were divided into four categories based on Non-HDL-C quartiles: Q1, < 2.35 mmol/L; Q2, 2.35–3.06 mmol/L; Q3, 3.07–3.83 mmol/L and Q4, > 3.83 mmol/L. As shown in Table 1, patients in the lowest quartile were more likely to be older, have a higher proportion of atrial fibrillation, have higher NIHSS scores and HDL-C compared with those in the highest quartile of Non-HDL-C. In addition, patients in the highest quartile were more likely to have a higher proportion of hypertension, diabetes mellitus, have higher SBP, DBP, glucose, TG, TC and LDL-C compared with those in the lowest quartile of Non-HDL-C.

**Table 1:** Baseline characteristics of participants according to Non-HDL-C quartiles

Characteristics	Quartiles of Non-HDL, mmol/l				*P*-value
	Q1: < 2.35	Q2: 2.35–3.06	Q3: 3.07–3.83	Q4: > 3.83	
	*n* = 514	*n* = 510	*n* = 511	*n* = 508	
Age, (Mean ± SD), years	66 ± 15	65 ± 14	65 ± 14	63 ± 14	< 0.001^a^
Male, n (%)	327 (63.6%)	341 (66.9%)	321 (62.8%)	301 (59.3%)	0.093^b^
Medical history					
Hypertension, n (%)	269 (52.3%)	265 (52.0%)	297 (58.1%)	301 (59.3%)	0.030 ^b^
Diabetes mellitus, n (%)	129 (25.1%)	109 (21.4%)	91 (17.8%)	138 (27.2%)	0.002 ^b^
Hyperlipidaemia, n (%)	14 (2.7%)	19 (3.7%)	17 (3.3%)	25 (4.9%)	0.293 ^b^
Atrial fibrillation, n (%)	97 (18.9%)	62 (12.2%)	44 (8.6%)	32 (6.3%)	< 0.001 ^b^
Smoking, n (%)	213 (41.4%)	220 (43.1%)	223 (43.6%)	208 (40.9%)	0.787 ^b^
Alcohol consumption, n (%)	142 (27.6%)	141 (27.6%)	142 (27.8%	135 (26.6%)	0.971 ^b^
TOAST classification					
Large-artery atherosclerosis, n (%)	151 (29.4%)	156 (30.6%)	179 (35.0%)	185 (36.4%)	< 0.001^b^
Small-artery occlusion, n (%)	84 (16.3%)	112 (22.0%)	132 (25.8%)	156 (30.7%)	
Cardioembolic, n (%)	150 (29.2%)	130 (25.5%)	97 (19.0%)	57 (11.2%)	
Undetermined aetiology, n (%)	108 (21.0%)	93 (18.2%)	87 (17.0%)	98 (19.3%)	
Other aetiology, n (%)	21 (4.1%)	19 (3.7%)	16 (3.1%)	12 (2.4%)	
NIHSS on admission, median (IQR)	6 (2–13)	6 (2–11)	5 (2–11)	5 (2–10)	0.002 ^c^
SBP, (Mean ± SD), mmHg	141 ± 22	144 ± 23	147 ± 24	151 ± 23	< 0.001^a^
DBP, (Mean ± SD), mmHg	82 ± 14	85 ± 14	87 ± 15	88 ± 15	< 0.001 ^a^
Glucose, (Mean ± SD), mmol/L	7.61 ± 2.92	7.67 ± 3.06	7.62 ± 2.96	8.98 ± 4.44	< 0.001^a^
Thrombolysis, n (%)	31 (6.0%)	22 (4.3%)	21 (4.1%)	33 (6.5%)	0.216 ^b^
Thrombectomy, n (%)	33 (6.4%)	24 (4.3%)	22 (4.3%)	23 (4.5%)	0.386 ^b^
Lipid profile					
TG, (Mean ± SD), mmol/L	1.11 ± 0.72	1.36 ± 0.84	1.74 ± 1.03	2.53 ± 1.88	< 0.001^a^
TC, (Mean ± SD), mmol/L	3.14 ± 0.55	3.97 ± 0.41	4.63 ± 0.41	5.81 ± 0.84	< 0.001^a^
HDL-C, (Mean ± SD), mmol/L	1.29 ± 0.41	1.25 ± 0.36	1.23 ± 0.36	1.19 ± 0.35	0.001 ^a^
LDL-C, (Mean ± SD), mmol/L	1.58 ± 0.40	2.33 ± 0.30	2.87 ± 0.36	3.79 ± 0.77	< 0.001^a^
Non-HDL, (Mean ± SD), mmol/L	1.86 ± 0.36	2.72 ± 0.20	3.40 ± 0.22	4.62 ± 0.78	< 0.001^a^

*Abbreviations*: *TOAST* The Trial of ORG 10172 in Acute Stroke Treatment, *NHISS* National Institutes of Health Stroke Scale, *SBP* Systolic blood pressure, *DBP* Diastolic blood pressure, *TG* Triglyceride, *TC* Total cholesterol, *LDL-C* Low-density lipoprotein cholesterol, *HDL-C* High-density lipoprotein cholesterol, *Non-HDL-C* Non-high-density lipoprotein cholesterol

a ANOVA test

b Chi-squared test

c Kruskal–Wallis test

Of the 2043 patients, 232 (11.4%) were identified as HT, of whom 34 (1.7%) were symptomatic HT. Incidence of HT was 14.0% in quartile 1, 11.5% in quartile 2, 12.3% in quartile 3, and 7.1% in quartile 4 for LDL-C (*P* = 0.003), and was 14.8% in quartile 1, 13.1% in quartile 2, 11.2% in quartile 3, and 6.3% in quartile 4 for Non-HDL-C (*P* < 0.001).

### Association of Non-HDL-C and LDL-C with HT, symptomatic HT

In the univariate analysis, age (*P* < 0.001), males (*P* < 0.001), atrial fibrillation (*P* < 0.001), smoking (*P* = 0.033), alcohol consumption (*P* = 0.049), TOAST classification (*P* < 0.001), NIHSS scores on admission (*P* < 0.001), SBP (*P* < 0.001), DBP (*P* = 0.048), thrombolysis (*P* < 0.001), thrombectomy (*P* < 0.001), TG (*P* < 0.001), TC (*P* = 0.003), HDL-C (*P* = 0.005), LDL-C (*P* < 0.001) and Non-HDL-C (*P* < 0.001) were significantly associated with HT (Table 2). In addition, only age (*P* = 0.017), atrial fibrillation (*P* < 0.001), NIHSS scores on admission (*P* < 0.001), TC (*P* = 0.047) and Non-HDL-C (*P* = 0.028) were significantly related to symptomatic HT (Additional file 1: Table S1).

**Table 2:** Univariate analysis to identify risk factors associated with haemorrhagic transformation in patients with acute ischaemic stroke

Characteristic	Total patients (*n* = 2043)	With HT (*n* = 232)	Without HT (*n* = 1811)	*P*-value
Age (Mean ± SD), years	65 ± 14	68 ± 14	64 ± 14	< 0.001^a^
Males, n (%)	1290 (63.1%)	113 (48.7%)	1177 (65.0%)	< 0.001^b^
Medical history, n (%)				
Hypertension, n (%)	1132 (55.4%)	115 (49.5%)	1017 (56.2%)	0.864^b^
Diabetes mellitus, n (%)	467 (22.9%)	52 (22.4%)	415 (22.9%)	0.528^b^
Hyperlipidaemia, n (%)	75 (3.7%)	7 (3.0%)	68 (3.8%)	0.574^b^
Atrial fibrillation, n (%)	235 (11.5%)	69 (29.7%)	166 (9.2%)	< 0.001^b^
Smoking, n (%)	864 (42.3%)	83 (35.8%)	781 (43.1%)	0.033^b^
Alcohol consumption, n (%)	560 (27.4%)	51 (22.0%)	509 (28.1%)	0.049^b^
TOAST classification				
Large-artery atherosclerosis, n (%)	671 (32.8%)	73 (31.5%)	598 (33.0%)	< 0.001^b^
Small-artery occlusion, n (%)	484 (23.7%)	4 (1.7%)	480 (26.5%)	
Cardioembolic, n (%)	434 (21.2%)	111 (47.8%)	323 (17.8%)	
Undetermined aetiology, n (%)	386 (18.9%)	40 (16.1%)	346 (19.3%)	
Other aetiology, n (%)	68 (3.3%)	4 (1.7%)	64 (3.5%)	
NIHSS on admission, median (IQR)	5 (2–10)	11(6–18)	5 (2–10)	< 0.001^c^
SBP, (Mean ± SD), mmHg	146 ± 23	140 ± 23	146 ± 23	< 0.001^a^
DBP, (Mean ± SD), mmHg	85 ± 15	84 ± 16	86 ± 15	0.048^a^
Glucose, (Mean ± SD), mmol/L	7.97 ± 3.45	8.16 ± 2.52	7.94 ± 3.55	0.233^a^
Thrombolysis, n (%)	107 (5.2%)	27 (11.6%)	80 (4.4%)	< 0.001^b^
Thrombectomy, n (%)	102 (5.0%)	24 (10.3%)	78 (4.3%)	< 0.001^b^
Lipid profile				
TG, (Mean ± SD), mmol/L	1.68 ± 1.32	1.37 ± 1.01	1.72 ± 1.35	< 0.001^a^
TC, (Mean ± SD), mmol/L	4.38 ± 1.13	4.18 ± 1.11	4.41 ± 1.14	0.003^a^
HDL-C, (Mean ± SD), mmol/L	1.24 ± 0.37	1.32 ± 0.44	1.23 ± 0.36	0.005^a^
LDL-C, (Mean ± SD), mmol/L	2.64 ± 0.95	2.44 ± 0.86	2.66 ± 0.95	< 0.001^a^
Non-HDL-C, (Mean ± SD), mmol/L	3.14 ± 1.11	2.86 ± 1.05	3.18 ± 1.11	< 0.001^a^

*Abbreviations*; *NHISS* National Institutes of Health Stroke Scale, *SBP* Systolic blood pressure, *DBP* Diastolic blood pressure, *TG* Triglyceride, *TC* Total cholesterol, *LDL-C* Low-density lipoprotein cholesterol, *HDL-C* High-density lipoprotein cholesterol. *Non-HDL-C* Non-high-density lipoprotein cholesterol

a Student’s *t* test

b Chi-squared test

c Mann-Whitney Test

Table 3 shows the association between quartiles of Non-HDL-C or LDL-C and HT. After adjusting for age and sex in model 1, patients in the lower Non-HDL-C quartiles were associated with increased risks of HT (*P* for trend < 0.001). Compared with the highest quartiles, the first, second and third quartiles of Non-HDL-C were associated with increased risk of HT (adjusted ORs 1.74 [95% CI 1.09–2.78], 2.01[95% CI 1.26–3.20], and 1.76 [95% CI 1.10–2.83], respectively) after adjusting for age, sex, NIHSS scores on admission, atrial fibrillation, smoking, alcohol consumption, SBP, thrombolysis, thrombectomy and TOAST classification in model 2. However, the only significant association was found between the third quartiles of Non-HDL-C and symptomatic HT (adjusted ORs 3.82 [95% CI 1.05–13.85]) after adjusting for age and NIHSS scores on admission (Additional file 2: Table S2).

**Table 3:** Association of quartiles of Non-HDL-C, LDL-C and haemorrhagic transformation

Variables	Crude			Model 1			Model 2		
	OR	95% CI	*P*-value	OR	95% CI	*P*-value	OR	95% CI	*P*-value
Non-HDL-C									
Q1: < 2.35 mmol/L	2.58	1.67–3.98	< 0.001	2.60	1.68–4.03	< 0.001	1.74	1.09–2.78	0.019
Q2: 2.35–3.06 mmol/L	2.25	1.45–3.50	< 0.001	2.34	1.50–3.66	< 0.001	2.01	1.26–3.20	0.003
Q3: 3.07–3.83 mmol/L	1.87	1.19–2.93	0.007	1.88	1.19–2.97	< 0.001	1.76	1.10–2.83	0.019
Q4: > 3.83 mmol/L	1.00			1.00			1.00		
Age (years)				1.01	1.00–1.02	0.006	1.01	1.00–1.02	0.136
Sex (female/male)				0.53	0.40–0.70	< 0.001	0.52	0.35–0.78	0.002
NIHSS							1.08	1.06–1.10	< 0.001
Atrial fibrillation							1.68	1.13–2.50	0.011
Smoking							1.54	0.99–2.38	0.054
Alcohol consumption							0.99	0.65–1.50	0.955
SBP (mmHg)							0.99	0.98–1.00	0.002
Thrombolysis							2.07	1.25–3.43	0.005
Thrombectomy							1.81	1.05–3.12	0.032
TOAST classification (cardioembolic/non cardioembolic)							2.07	1.45–2.94	< 0.001
LDL-C									
Q1: < 1.99 mmol/L	2.20	1.45–3.33	< 0.001	2.26	< 0.001	1.48–3.44	1.57	1.00–2.47	0.049
Q2: 1.99–2.56 mmol/L	1.70	1.10–2.62	0.017	1.77	0.011	1.14–2.75	1.51	0.95–2.40	0.079
Q3: 2.57–3.24 mmol/L	1.83	1.19–2.81	0.006	1.85	0.005	1.20–2.85	1.82	1.16–2.87	0.009
Q4: > 3.24 mmol/L	1.00			1.00			1.00		
Age (years)				1.02	1.00–1.03	0.004	1.01	1.00–1.02	0.129
Sex (female/male)				0.53	0.40–0.70	< 0.001	0.53	0.35–0.80	0.002
NIHSS							1.08	1.06–1.10	< 0.001
Atrial fibrillation							1.67	1.12–2.49	0.011
Smoking							1.53	0.99–2.36	0.056
Alcohol consumption							0.98	0.65–1.49	0.936
SBP (mmHg)							0.99	0.00–1.00	0.002
Thrombolysis							2.03	1.23–3.36	0.006
Thrombectomy							1.81	1.05–3.11	0.033
TOAST classification (cardioembolic/non cardioembolic)							2.13	1.50–3.02	< 0.001

*Abbreviations*: *OR* Odds ratio, *CI* Confidence interval, *Non-HDL-C* Non-high-density lipoprotein cholesterol, *LDL-C* Low-density lipoprotein cholesterol, *NHISS* National Institutes of Health Stroke Scale, *SBP* Systolic blood pressure, *TOAST* The Trial of ORG 10172 in Acute Stroke Treatment

After adjusting age and sex in model 1, patients in the lower LDL-C quartiles were associated with increased risks of HT (*P* for trend < 0.001). Compared with the highest quartiles, the first and third quartiles of LDL-C were associated with increased risks of HT (adjusted ORs 1.57 [95%CI 1.00–2.47] and 1.82 [95%CI 1.16–2.87]), but not the second quartiles (adjusted ORs 1.51 [95%CI 0.95–2.40]) after adjusting for age, sex, NIHSS scores on admission, atrial fibrillation, smoking, alcohol consumption, SBP, thrombolysis, thrombectomy and TOAST classification in model 2 (Table 3). No obvious relationship was found between LDL-C and symptomatic HT after adjusting for age and NIHSS scores on admission (Additional file 2: Table S2).

**Fig. 2:**
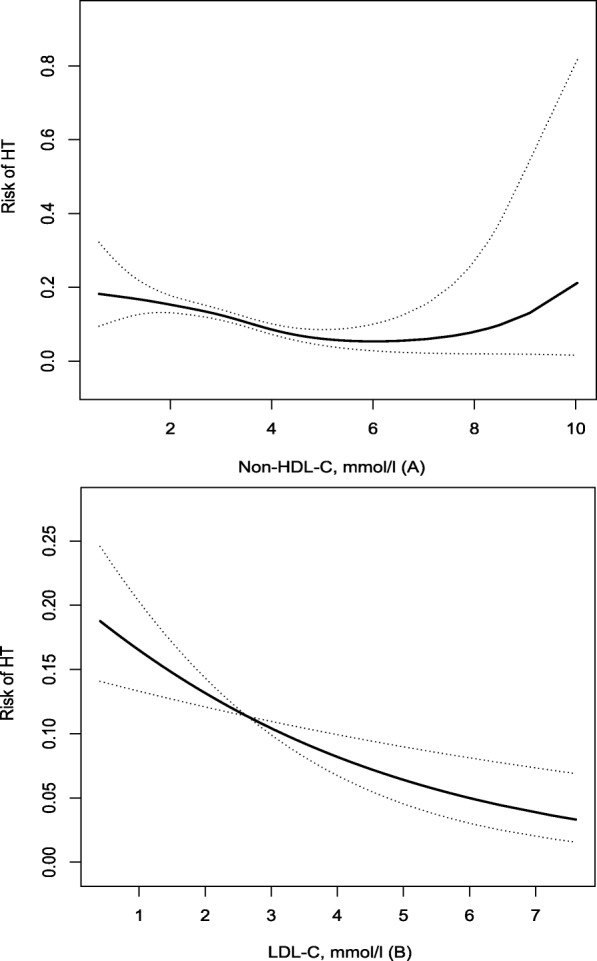
Relationship of Non-HDL-C (A), LDL-C (B) with risk of haemorrhagic transformation after acute ischaemic stroke. Risk of haemorrhagic transformation and 95% confidence intervals determined using the generalized additive model

Using a multiple-adjusted spline regression, no nonlinear trend was found between Non-HDL-C, LDL-C and HT (Fig. 2). When adding Non-HDL-C or LDL-C to model 2, the C-statistics were 0.79 (95%CI 0.77–0.80, *P* < 0.001) for Non-HDL-C and 0.78 (95%CI 0.77–0.80, *P* < 0.001) for LDL-C (Table 4). In addition, when adding Non-HDL-C, LDL-C to model 2 containing conventional risk factors significantly improved predictive ability (Non-HDL-C, net reclassification index 0.24, 95%CI 0.17–0.31, *P* < 0.001; LDL-C, net reclassification index 0.15, 95%CI 0.08–0.22, *P* = 0.03) (Table 4).

**Table 4:** C statistics and net reclassification index for HT by Non-HDL-C, LDL-C among patients with acute ischaemic stroke

Variable	C statistics (95%CI)	*P*-value	NRI (95%CI)	*P*-value
Non-HDL-C	0.59 (0.55–0.63)	< 0.001	NA	NA
LDL-C	0.57 (0.53–0.61)	0.001	NA	NA
Non-HDL-C + Model 2	0.79 (0.77–0.80)	< 0.001	0.24 (0.17–0.31)	< 0.001
LDL-C + Model 2	0.78 (0.77–0.80)	< 0.001	0.15 (0.08–0.22)	0.03

Model 2 adjusted for age, sex, National Institutes of Health Stroke Scale scores on admission, atrial fibrillation, smoking, alcohol consumption, systolic blood pressure, thrombolysis, thrombectomy and the Trial of ORG 10172 in Acute Stroke Treatment classification. NRI, net reclassification improvement

### Patients' age affects the relationship between non-HDL-C and HT

Age is an interaction factor between Non-HDL-C and HT(*P* = 0.021). Limiting the analysis to younger patients (< 60 years) showed a significant negative relationship between Non-HDL-C and HT (OR 0.64, 95%CI 0.47–0.87), *P* < 0.01), whereas this relationship was no longer significant in older patients (≥60 years) (Table 5). After adjustment for potential confounding variables, we found that the relationship between Non-HDL-C and HT did not change by sex, baseline NIHSS score, atrial fibrillation, smoking, alcohol consumption, SBP, reperfusion therapy (thrombolysis/thrombectomy) and stroke subtype (all *P* for interaction > 0.05) (Table 5).

**Table 5:** Stratified logistic regression analysis to identify variables that modify the correlation between Non-HDL-C and haemorrhagic transformation

	OR (95%CI), *P*-value^a^	*P* for interaction^a^
Age		
< 60	0.64 (0.47–0.87), P < 0.001	0.021
≥ 60	0.95 (0.81–1.12), 0.523	
Sex		
Male	0.78 (0.64–0.96) 0.021	0.188
Female	0.95 (0.78–1.15) 0.586	
Baseline NIHSS score		
< 15	0.90 (0.76–1.06) 0.217	0.363
≥ 15	0.78 (0.59–1.02) 0.069	
Atrial Fibrillation		
No	0.82 (0.69–0.96) 0.016	0.151
Yes	1.05 (0.78–1.40) 0.760	
Smoking		
No	0.89 (0.74–1.06) 0.190	0.655
Yes	0.83 (0.66–1.04) 0.109	
Alcohol consumption		
No	0.91 (0.78–1.07) 0.261	0.160
Yes	0.71 (0.53–0.97) 0.032	
SBP		
< 140	0.91 (0.74–1.11) 0.338	0.404
≥ 140	0.80 (0.66–0.98) 0.031	
Reperfusion therapy (Thrombolysis/Thrombectomy)		
No	0.86 (0.74–1.01) 0.070	0.944
Yes	0.88 (0.62–1.24) 0.452	
Stroke subtype		
Non-cardioembolic	0.79 (0.66–0.95) 0.013	0.134
Cardioembolic	0.99 (0.79–1.25) 0.953	

^a^Above model adjusted for age, sex, National Institutes of Health Stroke Scale scores on admission, atrial fibrillation, smoking, alcohol consumption, systolic blood pressure, thrombolysis, thrombectomy and Stroke subtype. In each case, the model is not adjusted for the stratification variable

## Discussion

In the present study, we found low Non-HDL-C was associated with an increased risk of HT in patients with AIS after adjustment for known risk factors. In addition, Non-HDL-C has similar effects as LDL-C for predicting HT.

Some studies showed that Non-HDL-C was a good biomarker for predicting cardiovascular events [7, 18, 19]. Despite these data, the role of the Non-HDL-C for HT is still not clear in patients with ischaemic stroke. In the present study, low Non-HDL-C was independently associated with increased risks of HT. In addition, no robust association was observed between LDL-C and HT. Prior studies [4, 20] have shown that low LDL-C was related to greater risk for HT; however, these studies included patients with ischaemic stroke receiving intravenous or intra-arterial rt-PA and mechanical recanalization. Conversely, other research [21–23] stated that LDL-C on admission was not associated with intracranial haemorrhage after intravenous thrombolysis. In our study, we found lower LDL-C was significantly related to higher risks of HT in the univariate analysis, but this association was attenuated after adjusting for risk factors, suggesting a possible mediating effect of unmeasured confounders. Further studies are needed to clarify the association especially in general AIS patients.

The mechanisms that explain the association of cholesterol and HT are uncertain, but there are some possible explanations as follows. First, cholesterol may play a great role in keeping the integrity of cerebral vascular vessel. It is reported that low level of cholesterol could cause the increased permeability of the erythrocyte membrane [24], and even contribute to the leakage and rupture of vessels wall [25]. Second, cholesterol is likely to affect aggregation of platelet. Some studies have shown that low level of cholesterol might lead to decreased platelet aggregation, and then increase the risk of bleeding [26]. Third, abnormal blood lipid levels could result in the increased plasma viscosity and whole blood viscosity, and then cholesterol would be accumulated in endothelium, thereby exciting the sympathetic nervous system and renin angiotensin system, with the injury of vascular wall [27, 28]. Further studies are needed to verify the mechanism between serum lipid levels and HT.

Although increasing evidence [8–10] indicated that Non-HDL-C was superior to LDL-C in terms of predicting the risk of cardiovascular disease, we found Non-HDL-C has similar predictive values as LDL-C for HT in AIS. The addition of Non-HDL-C or LDL-C to a conventional risk factor model could improve predictive ability for HT, suggesting that Non-HDL-C could be a potential predictive marker for HT as well as LDL-C. Furthermore, Non-HDL-C is more accurate and reliable when measured in the non-fasting sate compared with LDL-C [29]. In addition, some guidelines on the management of blood cholesterol has recommended that Non-HDL-C could be as a primary goal in the primary and secondary prevention of cardiovascular disease [30–32].

In this study, we found Non-HDL-C was negatively associated with HT in younger stroke patients. In older stroke patients, Non-HDL-C is also negatively related to HT although there was no significant difference. One possible explanation for the difference is that malnutrition is more commonly seen in elderly population [33] and malnutrition might lead to decreased serum cholesterol [34]. In addition, there are other factors that may contribute to the occurrence of HT. Older people usually present arterial stiffness that recently has been recognised as a possible risk factor for HT [35].

Interestingly, our study found that SBP on admission was significantly lower in patients with HT (140 ± 23 mmHg) than those without HT (146 ± 23 mmHg), which is in line with some previous studies [36–38]. A possible explanation might be that slightly elevated blood pressure could provide an adequate cerebral blood supply, and thereby reduce the damage to blood-brain barrier due to ischemia and hypoxia [39], which may prevent the occurrence of HT. A much-debated question is whether blood pressure is related to HT. Some studies [40–43] showed elevated SBP was associated with increased risks of HT, whereas other studies [44–46] did not observe the association. In addition, recent studies have reported that blood pressure variability, rather than the single measure of blood pressure on admission, is an emerging risk factor for HT [47–49]. More research is needed in this field.

Our study has some limitations. First, patients presented with HT at admission were excluded. Therefore, the results might not be generalizable to all ischaemic stroke patients. However, the proportion of patients with HT at admission is low (3.9%) in this study. Second, this study was just an observational, single-centre study, so the findings might not be generalized to the whole Chinese population. Third, although we struggled to obtain medical history, there might be some omissions. Therefore, the results of this study should be interpreted cautiously.

## Conclusions

In conclusion, low Non-HDL-C was independently associated with an increased risk of HT. In addition, Non-HDL-C has similar effects as LDL-C for predicting HT. These findings suggest that patients with low Non-HDL-C or LDL-C are prone to haemorrhagic transformation and those might be considered in practice to reduce the risk of haemorrhagic transformation. Further large sample size studies are needed to confirm these findings.

## Supplementary information

**Additional file 1: Table S1.** Univariate analysis to identify risk factors associated with symptomatic haemorrhagic transformation in patients with acute ischaemic stroke.

**Additional file 2: Table S2.** Association of quartiles of Non-HDL-C, LDL-C and symptomatic haemorrhagic transformation.

## Data Availability

The data used in this study are available from the corresponding author upon reasonable request.

## Abbreviations

AIS: Acute ischaemic stroke
CI: Confidence interval
CT: Computed tomography
DBP: Diastolic blood pressure
HT: Haemorrhagic transformation
LDL-C: Low-density lipoprotein cholesterol
MRI: Magnetic resonance imaging
NIHSS: National Institutes of Health Stroke Scale
Non-HDL-C: Non-high-density lipoprotein cholesterol
NRI: Net reclassification index
OR: Odds ratio
ORs: Odds ratios
SBP: Systolic blood pressure
TC: Total cholesterol
TG: Triglycerides
TOAST: The Trial of ORG 10172 in Acute Stroke Treatment
