# Supplementary material for: Association between non-high-density lipoprotein cholesterol and haemorrhagic transformation in patients with acute ischaemic stroke
Source: BMC Neurol. 2020 Feb 7;20:47. doi: 10.1186/s12883-020-1615-9 (PMC7007671; doi:10.1186/s12883-020-1615-9)
Supplement: Supplementary file 1 — Additional file 1: Table S1. Univariate analysis to identify risk factors associated with symptomatic haemorrhagic transformation in patients with acute ischaemic stroke. [file 12883_2020_1615_MOESM1_ESM.doc]

**Supplemental Table 1** Univariate analysis to identify risk factors associated with symptomatic haemorrhagic transformation in patients with acute ischaemic stroke

|  | No symptomatic HT (n=2009) | symptomatic HT (n=34) | P-value |
| --- | --- | --- | --- |
| Age (Mean ± SD), years | 65 ± 14 | 70 ± 13 | 0.017^a^ |
| Males, n (%) | 1267 (63.1%) | 23 (67.6%) | 0.583 ^b^ |
| Medical history, n (%) | | | |
| Hypertension, n (%) | 1112 (55.34%) | 20 (58.8%) | 0.686 ^b^ |
| Diabetes mellitus, n (%) | 461 (23.0%) | 6 (17.7%) | 0.543 ^d^ |
| Hyperlipidaemia, n (%) | 75 (3.73%) | 0 (0.00%) | 0.634 ^d^ |
| Atrial fibrillation, n (%) | 222 (11.1%) | 13 (38.2%) | <0.001^b^ |
| Smoking, n (%) | 851 (42.4%) | 13 (38.2%) | 0.629 ^b^ |
| Alcohol consumption, n (%) | 550 (27.4%) | 10 (29.4%) | 0.846 ^d^ |
| TOAST classification | | | |
| Large-artery atherosclerosis, n (%) | 660 (32.9%) | 11 (32.4%) | <0.001^b^ |
| Small-artery occlusion, n (%) | 484 (24.1%) | 0 (0%) |  |
| Cardioembolic, n (%) | 415 (20.7%) | 19 (55.9%) |  |
| Undetermined aetiology, n (%) | 383 (19.1%) | 3 (8.8%) |  |
| Other aetiology, n (%) | 67 (3.3%) | 1 (2.9%) |  |
| NIHSS on admission, median (IQR) | 5 (2-11) | 15 (9-20) | <0.00 ^c^ |
| SBP, (Mean ± SD), mmHg | 146 ± 23 | 144 ± 21 | 0.741^a^ |
| DBP, (Mean ± SD), mmHg | 85 ± 15 | 86 ± 14 | 0.599^a^ |
| Glucose, (Mean ± SD), mmol/L | 7.97 ± 3.46 | 8.12 ± 2.56 | 0.791^a^ |
| Thrombolysis, n (%) | 103 (5.1%) | 4 (11.8%) | 0.099 ^d^ |
| Thrombectomy, n (%) | 96 (4.8%) | 6 (17.7%) | 0.006 ^d^ |
| Lipid profile | | | |
| TG, (Mean ± SD), mmol/L | 1.69 ± 1.32 | 1.49 ± 1.03 | 0.38^a^ |
| TC, (Mean ± SD), mmol/L | 4.39 ± 1.14 | 4 ± 0.90 | 0.047^a^ |
| HDL-C, (Mean ± SD), mmol/L | 1.24 ± 0.37 | 1.27 ± 0.34 | 0.626^a^ |
| LDL-C, (Mean ± SD), mmol/L | 2.64 ± 0.95 | 2.33 ± 0.77 | 0.057^a^ |
| Non-HDL-C, (Mean ± SD), mmol/L | 3.15 ± 1.11 | 2.73 ± 0.86 | 0.028^a^ |

Abbreviations; NHISS, National Institutes of Health Stroke Scale; SBP; systolic blood pressure; DBP; diastolic blood pressure; TG; triglyceride; TC; total cholesterol; LDL-C; low-density lipoprotein cholesterol; HDL-C; high-density lipoprotein cholesterol. Non-HDL-C; non-high-density lipoprotein cholesterol.

a Student’s *t* test

b Chi-squared test

c Mann-Whitney Test

d Fisher’s exact test
