# Supplementary material for: Association between non-high-density lipoprotein cholesterol and haemorrhagic transformation in patients with acute ischaemic stroke
Source: BMC Neurol. 2020 Feb 7;20:47. doi: 10.1186/s12883-020-1615-9 (PMC7007671; doi:10.1186/s12883-020-1615-9)
Supplement: Supplementary file 2 — Additional file 2: Table S2. Association of quartiles of Non-HDL-C, LDL-C and symptomatic haemorrhagic transformation. [file 12883_2020_1615_MOESM2_ESM.doc]

Table 2 Association of quartiles of Non-HDL-C, LDL-C and symptomatic haemorrhagic transformation

|  | Unadjusted |  | Adjusted* |  |
| --- | --- | --- | --- | --- |
|  | OR (95%CI) | *P*-value | OR (95%CI) | *P*-value |
| Non-HDL-C |  |  |  |  |
| Q1 | 4.02 (1.13-14.34) | 0.032 | 2.78 (0.76-10.19) | 0.122 |
| Q2 | 2.34 (0.60-9.11) | 0.219 | 2.11 (0.54-8.34) | 0.285 |
| Q3 | 4.05 (1.14, 14.43) | 0.031 | 3.82 (1.05-13.85) | 0.041 |
| Q4 | 1 |  | 1 |  |
| *P* for trend | 0.069 | | 0.308 | |
| LDL-C |  |  |  |  |
| Q1 | 3.28 (0.90-11.99) | 0.072 | 2.38 (0.63-8.90) | 0.199 |
| Q2 | 3.74 (1.04-13.49) | 0.044 | 3.59 (0.98-13.18) | 0.054 |
| Q3 | 3.34 (0.91-12.20) | 0.068 | 3.40 (0.91-12.64) | 0.068 |
| Q4 | 1 |  | 1 |  |
| *P* for trend | 0.087 | | 0.316 | |

*Adjusted for age and National Institutes of Health Stroke Scale scores at admission.

Abbreviations; OR, odds ratio; CI, confidence interval; Non-HDL-C; non-high-density lipoprotein cholesterol; LDL-C; low-density lipoprotein cholesterol.
